# Various Formulæ for the Gelatinization of Cod-Liver Oil

**Published:** 1859-08

**Authors:** 


### VARIOUS FORMULÆ FOR THE GELATINIZATION OF COD-LIVER OIL.

#### M. STANISLAS MARTIN'S JELLY MODIFIED.

| | | |
|---|---|---|
| ℞. | Cod-liver oil, - - - | ℥ ij. |
| | Fresh spermaceti, - - | ʒ ijss. |
| | Simple syrup, - - - | ʒ vj. |
| | Jamaica rum, - - - | ʒ vj. |

Beat the ingredients together with the aid of heat, and when the mixture has acquired some consistence, pour it into a wide-mouthed bottle.

#### COD-LIVER OIL SOLIDIFIED WITH GELATINE.

| | | |
|---|---|---|
| ℞. | Pure gelatine, - - - | ℥ ss. |
| | Water, - - - - | ℥ iv. |
| | Simple syrup, - - - | ℥ iv. |
| | Cod-liver oil, - - - | ℥ viij. |
| | Aromatic essence, - - | q. s. |

Dissolve the gelatine in the boiling water, and add successively the syrup, the oil, and the aromatic essence; place the vessel containing the entire in a bath of cold water; whip the jelly for five minutes at most, and then pour it, while still fluid, into a wide-mouthed glass bottle, furnished with a cork, or with a pewter cap, or if a bottle be not at hand, into a porcelain or earthenware pot, which should be carefully closed.

#### COD-LIVER OIL GELATINIZED WITH CARRAGEEN OR IRISH MOSS.

| | | |
|---|---|---|
| ℞. | Fucus crispus, - - - | ℥ ss. |
| | Water, - - - - | ℥ xvij. |
| | Simple syrup, - - - | ʒ viij. |
| | Cod-liver oil, - - - | ʒ viij. |
| | Aromatic, - - - | q. v. |

Boil the carrageen in the water for twenty minutes; pass the decoction through flannel; concentrate it until it is reduced to four ounces by weight; add the syrup, the oil, and the aromatic; whip the mixture briskly, having first placed it in a cold bath, and pour it, while still a little warm, into the vessel intended to receive it. The syrup may be replaced by an equal quantity of Garus' elixir, mint or vanilla cream or rum, etc.

M. Sauvin proposes to combine cod-liver oil with Iceland moss.

LICHEN AND COD-LIVER OIL.

℞. Iceland moss jelly, - - ℥ iv.
Gelatine, - - - ℈ iv.
Hydrocyanated cod-liver oil (to which two drops of essence of bitter almonds have been added), - - - ℥ vj.

Prepare the Iceland moss jelly in the usual manner; melt the gelatine and pass it into the vessel which is to hold it; then add the cod-liver oil; stir the entire with a spatula, until the mixture be homogeneous and the jelly begins to congeal. Dose—two or three spoonsful daily.—*Bull. Gen. de Therap.—Dublin Hospital Gazette, Aug.* 15, 1857, *p.* 254.

